# The neuronal mechanisms underlying improvement of impulsivity in ADHD by theta/beta neurofeedback

**DOI:** 10.1038/srep31178

**Published:** 2016-08-12

**Authors:** Annet Bluschke, Felicia Broschwitz, Simon Kohl, Veit Roessner, Christian Beste

**Affiliations:** 1 Cognitive Neurophysiology, Department of Child and Adolescent Psychiatry, Faculty of Medicine of the TU Dresden, Germany; 2 Experimental Neurobiology, National Institute of Mental Health, Klecany Czech Republic

## Abstract

Neurofeedback is increasingly recognized as an intervention to treat core symptoms of attention deficit hyperactivity disorder (ADHD). Despite the large number of studies having been carried out to evaluate its effectiveness, it is widely elusive what neuronal mechanisms related to the core symptoms of ADHD are modulated by neurofeedback. 19 children with ADHD undergoing 8 weeks of theta/beta neurofeedback and 17 waiting list controls performed a Go/Nogo task in a pre-post design. We used neurophysiological measures combining high-density EEG recording with source localization analyses using sLORETA. Compared to the waiting list ADHD control group, impulsive behaviour measured was reduced after neurofeedback treatment. The effects of neurofeedback were very specific for situations requiring inhibitory control over responses. The neurophysiological data shows that processes of perceptual gating, attentional selection and resource allocation processes were not affected by neurofeedback. Rather, neurofeedback effects seem to be based on the modulation of response inhibition processes in medial frontal cortices. The study shows that specific neuronal mechanisms underlying impulsivity are modulated by theta/beta neurofeedback in ADHD. The applied neurofeedback protocol could be particularly suitable to address inhibitory control. The study validates assumed functional neuroanatomical target regions of an established neurofeedback protocol on a neurophysiological level.

As one of the most prevalent neuropsychiatric disorders of childhood and adolescence^1^, Attention Deficit Hyperactivity Disorder (AD(H)D) is characterised by the three core symptoms of inattention, hyperactivity and increased impulsivity. The current consensus suggests a multimodal treatment approach for AD(H)D^2^. Combining aspects from a number of treatment modalities, neurofeedback as an intervention for AD(H)D has largely grown in popularity. During neurofeedback, patients are required to regulate their own cortical activity which is recorded using EEG electrodes and is presented to them via sounds or simple animations. Training is usually supplemented by elements of cognitive-behavioural therapy. While slow cortical potential (SCP) training addresses the ability to switch between cortical excitation and inhibition, frequency band neurofeedback trains patients with AD(H)D to down-regulate central theta activity while simultaneously increasing beta power, thus overall reducing the theta/beta ratio. This training is based on findings suggesting that children with AD(H)D are characterised by heightened theta and reduced beta power^3^,^4^,^5^. This has been interpreted as a sign of cortical hypoarousal and as reflecting an unprepared and inefficient cortical state compared to healthy controls^6^,^7^,^8^,^9^. Although this interpretation of the theta and beta oscillations has been criticised^10^,^11^ and it has been suggested that their power may not actually be altered in patients with AD(H)D^12^, a large number of studies, including randomised controlled trials and meta-analyses^13^, have demonstrated beneficial effects of neurofeedback for AD(H)D symptomatology^4^,^8^,^19^. However, its limitations and the influence of various additional treatment factors have been addressed as well^16^,^18^.

Despite the large amount of research that has so far been conducted on neurofeedback, it is, with only very few exceptions^20^,^21^, currently widely elusive what neuronal mechanisms related to core symptoms of AD(H)D are modulated by neurofeedback interventions. Studies examining neurophysiological changes have focussed on mechanisms that were actually trained during the respective neurofeedback protocols^20^,^22^. For a broader assessment of neurofeedback effects it is necessary to consider neurophysiological processes that are not equal to the trained neurofeedback parameters and reflect AD(H)D symptomatology on a neurophysiological level. Of particular interest here is the core symptom of impulsivity, which is closely related to the executive function of behavioural inhibition, a process frequently impaired in AD(H)D^25^,^26^. Impulsivity has recently been shown to be modulated by a number of dissociable cognitive-neurophysiological subprocesses^27^. Both basic perceptual processes and mechanisms related to response selection seem to play a crucial role for impulse control. Inhibition deficits can arise due to problems at the stage of perceptual and attentional selection^28^ or at that of response control^31^. It has been shown that impulsive behaviour in patients with AD(H)D is related to deficits on a multitude of stages of the processing cascade^32^ and it is now important to examine whether it is possible to influence these mechanisms through neurofeedback. The different subprocesses can be examined using event-related potentials (ERPs) in a Go/Nogo paradigm. In particular, early processes of perceptual gating and attentional selection are reflected by the parieto-occipital P1 and N1 components^33^, whereas resource allocation mechanisms are related to P2^34^. On the level of response selection, the frontal N2 during Nogo trials may reflect conflict monitoring or pre-motor inhibition processes, while the Nogo-P3 may reflect the inhibition process per se or the evaluation of response inhibition^35^. As theta/beta neurofeedback opts to modulate medial-central activations^40^, it is likely that neurofeedback will mainly affect processes reflected by the central Nogo-N2 and Nogo-P3 rather than more basic mechanisms related to perceptual/attentional gating or resource allocation. Ultimately, this study shows that indeed, specific neurophysiological mechanisms in medial frontal cortices are modulated by neurofeedback and are connected to reduced impulsive behaviours in paediatric patients with AD(H)D.

## Results

### Clinical data

Analysis of parental ADHD symptom rating using the Conners-3 scales indicated a significant interaction of *Group* and *Time point* across all six scales (F(1, 22) = 5.9, p = 0.02, η_p_^2^ = 0.2). After neurofeedback, AD(H)D symptoms overall were rated to be significantly lower (63.3 ± 1.9) than at pre-testing (66.3 ± 1.7) (p = 0.04). In the waiting list controls, no such differences between pre- (62.2 ± 3.8) and post-testing (64.9 ± 3.7) were found (p = 0.23). Considering the two most relevant subscales separately, we found a significant improvement on the Inattention subscale in the neurofeedback group (pre: 72.3 ± 9.6, post: 66.9 ± 10.8, p = 0.03) but not in the waiting list controls (pre: 68.3 ± 13.4, post: 68.9 ± 12.6, p = 0.82). Considering the Hyperactivity/Impulsivity subscale, the improvement in the neurofeedback group (pre: 70.2 ± 9.8, post: 65.9 ± 11.2) was found to be at trend level (p = 0.08). No effect (p = 0.15) was found for the waiting list controls (pre: 61.6 ± 15.8, post: 67.0 ± 15.2).

### Behavioural data

Behavioural data of one neurofeedback participant were lost due to technical error. We found a significant interaction between *Group* and *Time point* (F(1, 33) = 4.1, p = 0.05, η_p_^2^ = 0.1). After neurofeedback, patients with AD(H)D committed significantly fewer false alarms (32.1% ± 5.4 (m ± SD)) in Nogo trials than at pre-testing (43.9% ± 5.4) (F(1, 17) = 12.1, p = 0.003, η_p_^2^ = 0.42), indicating reduced impulsivity in this group. In the waiting list control group, no such differences between pre- (53.6% ± 5.6) and post-testing (52.8% ± 5.5) were found (F(1, 16) = 0.12, p = 0.74, η_p_^2^ < 0.01). The main effects of *Group* (F(1, 33) = 4.38, p = 0.04, η_p_^2^ = 0.12) and *Time Point* (F(1, 33) = 6.5, p = 0.02, η_p_^2^ = 0.16) were also significant. Waiting list controls (53.5% ± 5.3) generally committed more Nogo false alarms than neurofeedback participants (38.2% ± 5.1).

In terms of correct responses to Go stimuli, no significant differences were found between time points for either the neurofeedback group (pre: 94.5% ± 1.2, post: 95.9% ± 1.7) or the waiting list controls (pre: 96.0% ± 1.3, post: 95.5 ± 1.7) (Group^*^Time Point: F(1, 33) = 1.17, p = 0.29, η_p_^2^ = 0.03). There also were no main effects of *Group* (F(1, 33) = 0.09, p = 0.76, η_p_^2^ < 0.01) or *Time Point* (F(1, 33) = 0.3, p = 0.59, η_p_^2^ < 0.01). Similarly, reaction times (RTs) in Go trials did not differ significantly when compared before (neurofeedback: 466 ± 85 ms; waiting list: 428 ± 84 ms) and after the 8 weeks of participation (neurofeedback: 446 ± 55 ms, waiting list: 424 ± 75 ms) (*Group* * *Time Point*: F(1, 33) = 0.68, p = 0.41, η_p_^2^ = 0.02). There were also no main effects of *Group* (F(1, 33) = 1.75, p = 0.20, η_p_^2^ = 0.05) or *Time Point* (F(1, 33) = 1.01, p = 0.31, η_p_^2^ = 0.03). There were no differential effects concerning the first half and second half of the experiment (i.e. before and after the break) (all F < 0.5; p > 0.05).

### Neurophysiological data

#### Perceptual gating (P1) and attentional selection (N1)

**Figure 1:**
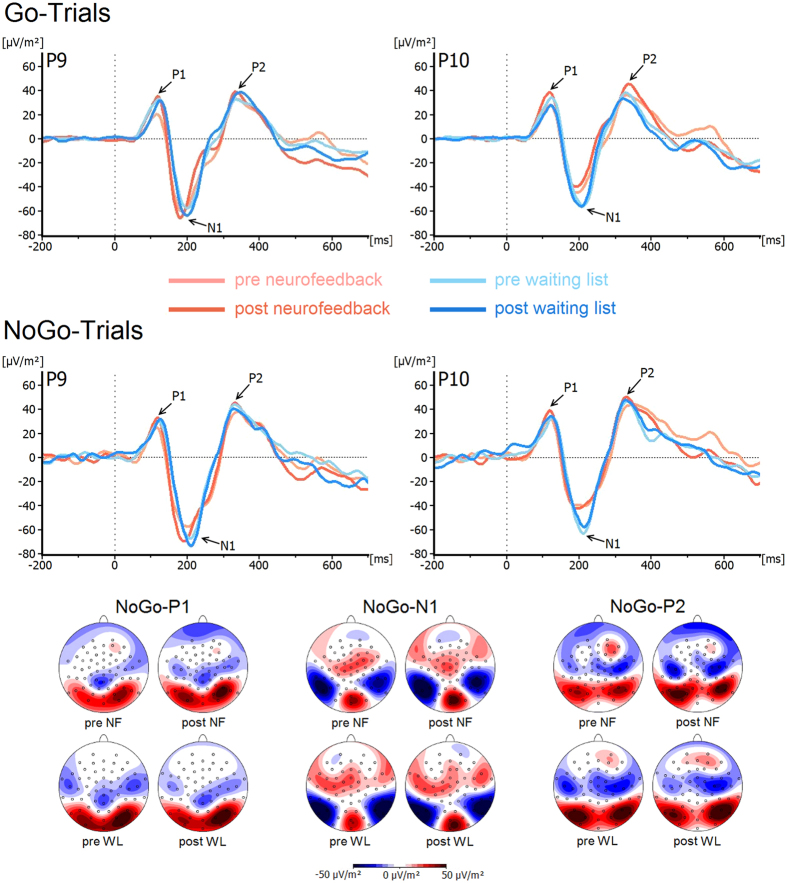
Stimulus-locked waveforms (current source density) and topographic maps for P1, N1 and P2 components, depicted for Go and Nogo trials, both experimental groups (NF = neurofeedback, WL = waiting list controls) and both time points at electrodes P9 and P10. Point 0 denotes Go/Nogo stimulus onset. In the Nogo-topographic maps blue denotes negative deflections whereas red reflects positive ones.

P1 and N1 components for both groups and for Go and Nogo trials are shown in Fig. 1. P1-analyses showed no main effect of *Group* (F(1, 34) = 0.02, p = 0.89, η_p_^2^ < 0.01) or *Time Point* (F(1, 34) = 0.58, p = 0.45, η_p_^2^ = 0.02) on P1 amplitudes and there was also no interaction between Group^*^Time Point (F(1, 34) = 0.65, p = 0.42, η_p_^2^ = 0.02) (neurofeedback_pre_: 25.6 ± 6.1 μV/m^2^, neurofeedback_post_: 35.2 ± 6.6 μV/m^2^; waiting list_pre_: 29.6 ± 6.5 μV/m^2^, waiting list_post_: 29.3 ± 6.9 μV/m^2^). This was also the case for all other main effects and interactions (all F < 1.5, all p > 0.23, all η_p_^2^ < 0.04).

Analyses of N1 amplitude revealed a main effect of *Electrode* (F(1, 34) = 9.3, p = 0.004, η_p_^2^ = 0.22) with more negative amplitudes over the left- (P9: −56.9 ± 5.4 μV/m^2^) compared to the right-sided electrode (P10: −42.2 ± 4.2 μV/m^2^). There were no significant differences between the neurofeedback group (pre: −48.7 ± 7.6 μV/m^2^, post: −52.7 ± 6.5 μV/m^2^) and the waiting list controls (pre: −47.7 ± 8.1 μV/m^2^, post: −49.2 ± 6.7 μV/m^2^) at either of the two time points (Group^*^Time Point: F(1, 34) = 0.43, p = 0.84, η_p_^2^ < 0.01). There were no other main effects or interactions (all F < 1.9, all p > 0.17, all η^2^ < 0.06).

For the P1 and N1, there were no differential effects concerning the first half and second half of the experiment (i.e. before and after the break) (all F < 0.6; p > 0.05).

#### Resource allocation (P2)

P2 components for both groups and for Go and Nogo trials are shown in Fig. 1. Concerning the amplitudes, there was a significant main effect *Go/Nogo* (F(1, 34) = 6.4, p = 0.02, η_p_^2^ = 0.16), showing significantly more positive amplitudes in Nogo- (24.1 ± 3.7 μV/m^2^) than in Go-trials (19.2 ± 3.4 μV/m^2^). The main effect of *Group* was not significant (F(1, 34) = 0.9, p = 0.35, η_p_^2^ = 0.03). There was no interaction of Group*Time Point (F(1, 34) = 1.04, p = 0.32, η_p_^2^ = 0.03) (neurofeedback_pre_: 19.2 ± 4.8 μV/m^2^, neurofeedback_post_: 17.6 ± 5.6 μV/m^2^) and the waiting list controls (waiting list_pre_: 22.3 ± 5.0 μV/m^2^, waiting list_post_: 27.5 ± 6.0 μV/m^2^). There were no other significant main effects or interactions in terms of amplitudes or latencies (all F < 0.9, all p > 0.33, all η^2^ < 0.03). For the P2 there was no differential effect concerning the first half and second half of the experiment (i.e. before and after the break) (all F < 0.45; p > 0.05).

#### Response selection processes (N2 and P3)

**Figure 2:**
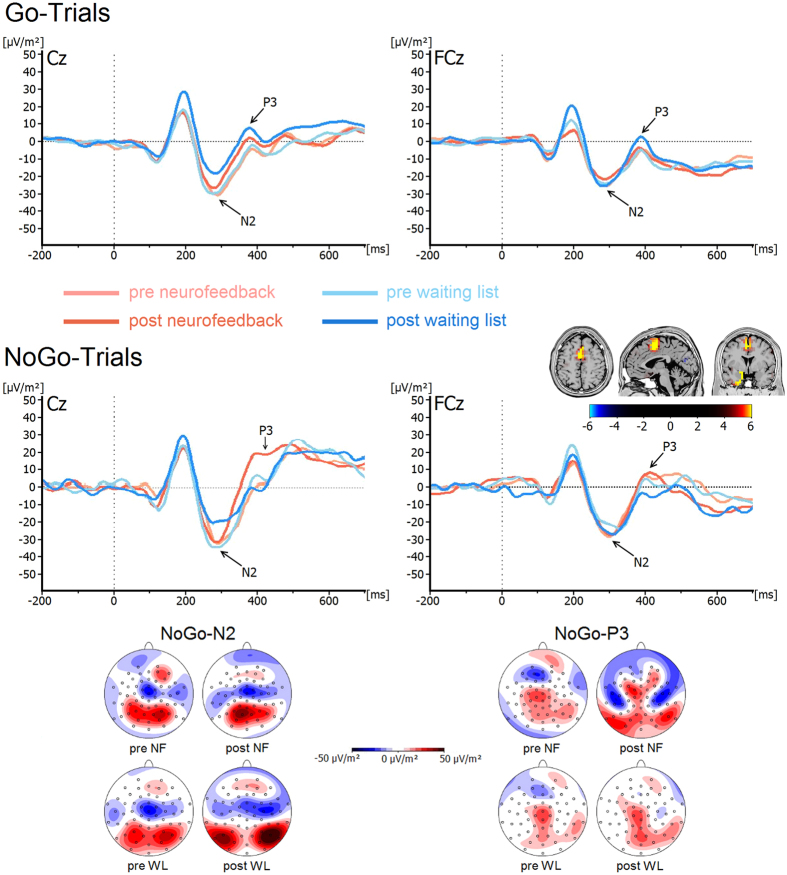
Stimulus-locked waveforms (current source density) and topographic maps for the N2 and P3 component, depicted for Go and Nogo trials, both experimental groups (NF = neurofeedback, WL = waiting list controls) and both time points at electrodes Cz and FCz. Point 0 denotes Go/Nogo stimulus onset. In the Nogo-topographic maps blue denotes negative deflections whereas red reflects positive ones. The sLORETA plot shows the pre-post difference in Nogo-P3 amplitudes within the ADHD group. Colours denote t-values corrected using randomization tests.

N2 and P3 components for both groups and for Go and Nogo trials are shown in Fig. 2.

The analyses of N2 amplitude and latencies revealed no main effects or interactions (all F < 3.1, all p > 0.09, all η_p_^2^ < 0.08) (neurofeedback_pre_: −28.5 ± 5.5 μV/m^2^, neurofeedback_post_: −26.0 ± 6.6 μV/m^2^; waiting list_pre_: −28.6 ± 5.8 μV/m^2^, waiting list_post_: −17.8 ± 6.9 μV/m^2^).

The analysis revealed a significant interaction of Time Point * Go/Nogo * Group (F(1, 34) = 4.1, p = 0.05, η_p_^2^ = 0.1). To analyze this interaction further, we examined each group separately for an interaction of Time Point * Go/Nogo. This interaction was only significant for the neurofeedback group (F(1, 18) = 4.2, p = 0.05, η_p_^2^ = 0.2), but not in the waiting list controls F(1, 18) = 1.2, p = 0.3, η_p_^2^ = 0.07). For the neurofeedback group there were no differences between time points on Go trials (pre neurofeedback: −4.6 ± 4.3 μV/m^2^, post neurofeedback: 1.4 ± 3.9 μV/m^2^) (t(18) = −1.12; p = 0.130), but only on Nogo trials (pre: −2.0 ± 7.5 μV/m^2^, post: 17.2 ± 8.5 μV/m^2^) (t(18) = −3.15; p = 0.005). On Nogo trials only, the P3-amplitude was more pronounced after 8 weeks of neurofeedback. The sLORETA analysis showed that the differences in Nogo-P3 amplitudes between the time points were due to activation differences in the anterior cingulate cortex (ACC) and superior frontal cortex (SFG) (BA24, BA6). There were no other statistically significant main effects or interactions in the overall ANOVA (F < 3.6, all p > 0.07, all η_p_^2^ < 0.09). In the neurofeedback group the P3 peak occurred approximately 100 ms earlier after training than before and when compared to the waiting list controls. There were no differential effects on N2 and P3 parameters concerning the first half and second half of the experiment (i.e. before and after the break) (all F < 0.6; p > 0.05).

## Discussion

In the current study, we examined what neurophysiological mechanisms underlying impulsive behaviour in paediatric patients with AD(H)D are modulated by 8 weeks of theta/beta neurofeedback training. The study provides evidence for the effectiveness of theta/beta neurofeedback training and goes beyond previous results that evaluated neurofeedback by examining parent and teacher ratings^14^,^15^,^41^. To improve on this, the current study employed rigorous experimental behavioural and neurophysiological measures combining high-density EEG recordings with source localization analyses using sLORETA. The results show that, compared to a waiting list control group, AD(H)D symptoms as measured by parent ratings were reduced after neurofeedback. Most importantly, this was also the case for experimentally measured impulsive behaviours. These were reduced after neurofeedback treatment as indicated by the lowered rate of Nogo false alarms. Here, effect sizes were even stronger than those obtained based on the parent ratings. Similarly, previous results also point towards an effectiveness of neurofeedback for impulse control^42^. The effects of neurofeedback were very specific for situations requiring inhibitory control over responses, since no effects were seen for RTs or missed Go trials. These behavioural results already suggest that cognitive subprocesses directly involved in inhibitory control are strongly modulated by the applied neurofeedback protocol and that this is less so the case for processes related to more basic attentional mechanisms.

This is exactly what is reflected by the neurophysiological data, providing insights into the neuronal mechanisms behind the beneficial effects of the applied neurofeedback protocol.

The ERP data shows that there were no effects on P1, N1 and P2 amplitudes or latencies in either of the groups. This suggests that processes of perceptual gating and attentional selection (P1 and N1 ERPs) (e.g. ref. 33, as well as resource allocation processes (P2 ERP)^34^,^43^ are not influenced by the theta/beta neurofeedback intervention as applied in this study^40^. This is plausible since these processes have frequently been shown to depend on parieto-occipital functional neuroanatomical structures^30^,^33^ and less so on medial central or frontal structures which, in contrast, have been shown to be important for response inhibition processes (for review: ref. 31). In this regard the EEG data shows that dissociable response inhibition subprocesses are differentially altered by neurofeedback treatment. It was the Nogo-P3 and not the Nogo-N2 that was altered by neurofeedback, suggesting that even at the response selection and inhibition level, the applied protocol has very specific effects and does not modulate conflict monitoring, but instead specifically affects response inhibition^35^,^40^. The source localization analysis suggests that areas in the medial frontal cortex, encompassing the ACC and SFG, are modulated. These areas have previously been shown to generate the Nogo-P3^35^,^41^.

As such this data shows, for the first time, that the applied neurofeedback protocol does indeed modulate medial frontal areas that are crucially involved in cognitive processes underlying core symptoms of AD(H)D – like impulsivity. It is possible that this overlap between the functional neuroanatomical networks being targeted during neurofeedback and those important for response control processes during inhibition is crucial for the effects to emerge. The entire pattern of results suggests that the applied neurofeedback protocol, which is currently widely used in AD(H)D treatment^40^, seems to specifically modulate response inhibition processes as one major facet of AD(H)D symptoms and less so attentional selection processes. However, the latter are central to inattention as another core deficit in AD(H)D and have frequently been reported to improve after theta/beta neurofeedback^13^. In the current study, any improvements in attention on the experimental level may have been masked by the fact that the current design focuses on bottom-up attentional processes. In contrast, attention as measured by behaviour ratings may reflect top-down attentional control processes that are more apparent to the raters. It is important to consider that behaviorally constructed inattention cannot always be equated to neurophysiologically constructed inattention. This is also reflected by the fact that on the symptom level, improvements in the neurofeedback group appeared to be stronger in regards to inattention than when considering hyperactivity/impulsivity. This lies in accordance with prior studies and meta-analyses which have found the effects of neurofeedback to be strongest for inattention^4^. In the current study, no such differences in AD(H)D symptoms were found in the waiting list controls.

These findings needs to be considered very carefully, since complete pre- and post-datasets were only available for a subset of the waiting list controls. A possible clinical implication of this result might thus be that the applied neurofeedback protocol could be particularly suitable for AD(H)D patients showing prominent impulsivity on a neuropsychological level rather than solely based on parent reports. A limitation of the study is that there was no randomized allocation of patients to the study groups. Furthermore, there was also no active control group, which would be important for evaluating the specificity of the intervention and the influence of unspecific treatment factors. However, by using objective performance and neurophysiological measures to evaluate neurofeedback effects, we were able to make the results largely independent of such unspecific treatment factors. It may also be argued that the sample size and the number of neurofeedback sessions was limited and that the data on AD(H)D symptoms was incomplete. However, as it is already known from randomised controlled trials and meta-analyses that neurofeedback is effective on the symptom level^10^,^13^,^15^,^16^,^42^, this is not too central for the current study for which the goal was to examine the underlying neurophysiological mechanisms. Also, the effects obtained were nevertheless quite strong, showing that the study was sufficiently powered and results are reliable. Due to the relatively small sample it was also not possible to examine whether the effect of neurofeedback was different for children with high vs. low impulsivity scores. However, this study used a classical neurofeedback protocol reducing theta frequency oscillations and increasing beta frequency oscillations. In it has been suggested that this protocol may not be optimal for affecting executive control processes because it ignores recent advances on the functional relevance of theta oscillations for cognitive control processes^40^. The current positive findings may thus largely be based on the upregulation of beta oscillations^46^ and/or complex interactive effects with theta modulations.

In summary, the study shows that impulsivity is positively affected by theta/beta neurofeedback training in AD(H)D. This is the first study showing what neuronal mechanisms are modulated by neurofeedback and are connected to reduced impulsivity. Further, we showed that specific neurophysiological mechanisms, that are directly associated with the process of response inhibition in medial frontal cortices, are modulated by theta/beta neurofeedback. The study therefore validates assumed functional neuroanatomical target areas of an established neurofeedback protocol on a neurophysiological level. Processes like perceptual gating, attentional selection and resource allocation during response inhibition were not modulated by neurofeedback. It needs to be tested in future studies to what extent the currently applied neurofeedback protocol can be modified to affect other facets of AD(H)D beyond impulsivity.

## Materials and Methods

### Patients and controls

Only patients in whom AD(H)D diagnoses had been determined according to standard clinical procedures (incl. parent and child interview, teacher report, symptom questionnaires, IQ testing, exclusion of underlying somatic disorders via EEG, ECG, audiometry and vision testing) were included in the study. All participants fulfilled criteria for AD(H)D according to ICD-10 criteria (F90.0, F90.1 or F98.8) and were regular patients of the outpatient clinic of the Department of Child and Adolescent Psychiatry, TU Dresden. Patients in whom additional severe or acute psychiatric (e.g. autism, tics, depressive episode) or somatic comorbidities had been diagnosed within the clinical care setting were excluded from the study. 19 patients (18 male, 11.2 ± 2.0 years, age range between 8 and 14 years, IQ: 97.2 ± 2.6), whose parents/legal guardians had contacted the study team after seeing advertisements about neurofeedback, participated in an 8 weeks theta/beta neurofeedback training. 9 of them were taking AD(H)D medication (immediate or extended release methylphenidate or atomoxetine). 17 patients were recruited to be part of the waiting list control group (all male, 11.3 ± 2.1 years, IQ: 103.5 ± 3.3, age range between 9 and 13 years). 9 of them were taking medication (immediate or extended release methylphenidate or atomoxetine). 5 of the patients in the neurofeedback group and 4 waiting list controls could not be tested in an unmedicated state. The two groups were recruited simultaneously and did not differ regarding age (t(34) = −0.12, p = 0.91), IQ (t(34) = −1.5, p = 0.14) and AD(H)D symptomatology. In the AD(H)D Symptom Checklist^43^, parents rated (0: no problems, 3: severe problems) their children in regards to inattention (average raw score neurofeedback group: 1.8 ± 0.15, waiting list controls: 1.9 ± 0.15, (t(34) = −0.95, p = 0.35)), hyperactivity (average raw score neurofeedback group: 1.2 ± 0.15, waiting list controls: 1.0 ± 0.19, (t(34) = 0.54, p = 0.6)) and impulsivity (average raw score neurofeedback group: 1.7 ± 0.16, waiting list controls: 1.7 ± 0.18, (t(34) = −0.04; p = 0.97), thus confirming AD(H)D symptomatology. Baseline ratings in the Conners-3 parent scale used for the pre-post assessment of ADHD symptomatology (see Results section) also revealed no significant differences between the two groups in any of the subscales (all t(34) <1.7; all p > 0.09). All subjects and their parents or legal guardians provided informed written consent according to the Declaration of Helsinki and the study was approved by the local ethics committee of the Medical Faculty of the TU Dresden.

### Neurofeedback protocol

Theta/beta-ratio neurofeedback training took place in two weekly sessions (one hour each) across 8 weeks. During the course of the training, patients were trained to downregulate theta power (4–8 Hz) and to upregulate beta power (13–20 Hz) recorded over electrode Cz. Eye movement data was recorded below the left eye for artefact correction, the left mastoid was used for referencing and the ground electrode was placed on the forehead. Theta and beta frequency ranges were made visible to participants via a custom-made software (“Self-regulation and Attention Management” (“SAM”), University of Erlangen, ref. 22). Time intervals containing artifacts occurring as a result of excessive movement were removed online. When this was the case, children were shown an unhappy smiley face, reminding them to reduce movements. After a two-minute baseline recording, children were able to move a cartoon character or car on the screen by regulating the frequencies in the desired direction. Within the animation, frequencies were also shown to the children via moving bars on the screen. During each session, around 3–6 neurofeedback blocks were conducted, each lasting from 5–10 minutes. From session 4–5 onwards, one or two of the blocks were conducted as transfer blocks in which children were given a task (e.g. concentration games, reading, school work) and were required to perform it without directly seeing feedback on the screen. After every training block (with immediate and delayed feedback), performance was reviewed with the participant. In adherence to standard protocols^15^,^22^, neurofeedback training was supplemented by elements of behavioural therapy, including psychoeducation, the development of attentional strategies, homework and a token system. To evaluate changes in AD(H)D symptoms, parents completed the parent scale of the Conners-3 questionnaire^48^. For the pre-post comparison, we analysed the six subscales (Inattention, Hyperactivity/Impulsivity, Learning Problems, Executive Functions, Aggressiveness and Peer Relations) in one ANOVA and additionally included the factors *Time Point* (pre vs. post) and the between-subjects factor *Group* (Neurofeedback vs. waiting list controls). Complete clinical data was available for 16 of the patients in the neurofeedback group and for 8 of the waiting list controls. The remaining questionnaires were missing since parents were unable to return completed questionnaires.

### Task

A standard Go/Nogo task was used to examine response inhibition performance^32^,^44^ before and after 8 weeks of neurofeedback or waiting list status^27^. In the task, one out of two words was presented (300 ms) on a monitor: ‘DRÜCK’ (German for ‘PRESS’; Go stimulus) and ‘STOP’ (German for ‘STOP’; Nogo stimulus). Participants were asked to respond fast (i.e. within 500 ms) on the ‘DRÜCK’ stimulus and refrain from responding on the ‘STOP’ stimulus. The subjects had to react with the right index finger. The inter-trial interval (ITI) was jittered between 1600 ms and 1800 ms. The experiment consisted of 248 Go trials and 112 Nogo trials presented in a pseudo-randomized order to avoid consecutive identical trial conditions. The task lasted approximately 20 minutes with a break in between after 10 minutes.

### EEG recording and analysis and source reconstruction

The EEG was recorded with an equidistant electrode setup from 60 Ag/AgCl electrodes with a sampling rate of 500 Hz (reference at Fpz, ground electrode at θ = 58, ф = 78). Electrode impedances were kept below 5 kΩ. Data processing took place analogous to the procedure described in ref. 27: During off-line data processing, the recorded data was down-sampled to 256 Hz and a band-pass filter (0.5–20 Hz, slope: 48 db/oct) was applied. Technical artifacts were removed during the manual inspection of the raw data. An independent component analysis was subsequently used to detect and remove periodically occurring artifacts (pulse artifacts, horizontal and vertical eye movements). Data was segmented to the onset the Go and Nogo stimuli (−200 ms −1500 ms). Only trials with correct responses on Go and without responses on Nogo trials were analysed further. Remaining artifacts were removed using an automatic artefact rejection procedure with an amplitude criterion (maximal amplitude: 200 μV, minimal amplitude: −200 μV) and using a maximal value difference of 200 μV in a 200 ms interval as well as an activity below 0.5 μV in a 100 ms period as rejection criteria. A current source density transformation was used to allow a reference-free evaluation of the EEG data which helps to find the electrodes showing the strongest effects^50^. Data were then baseline corrected to a time interval from −200 ms to 0 ms and segments were averaged for each condition. Single-subject ERP-amplitudes were quantified as the mean amplitude in a defined time interval. The following electrodes were chosen for ERP quantification (i.e. peak amplitudes and corresponding peak latencies) on the basis of the scalp topography: The P1 component was measured over P9 and P10 (110–130 ms). The N1 component was measured over electrodes P9 and P10 (175–195 ms). P2 amplitudes were exported from electrodes Cz and FCz (190–210 ms). Electrodes FCz and Cz were also used to measure the N2 (260–320 ms) and P3 components (380–400 ms). This choice of electrodes and time windows was validated using a statistical procedure described in Mückschel *et al*.^46^. This validation procedure was confined to the Nogo trials, since these are of interest in the present study. This validation procedure revealed the same electrodes and time windows as identified by visual inspection. To examine what functional neuroanatomical networks are modulated by neurofeedback intervention, we conduct source localization using sLORETA (standardized low resolution brain electromagnetic tomography^47^. sLORETA provides a single solution to the inverse problem^52^,^53^. For sLORETA, the intracerebral volume is partitioned into 6239 voxels at 5 mm spatial resolution and then the standardized current density at each voxel is calculated in a realistic head model based on the MNI152 template. It has been mathematically proven that sLORETA provides reliable results and there is evidence from EEG/fMRI and neuronavigated EEG/TMS studies underlining the validity of the sources estimated using sLORETA^53^,^54^. In this study we restricted the sLORETA analysis to effects in ERPs that show the specific effect of the neurofeedback intervention, i.e. for ERPs that show a significant *Group* * *Time Point* interaction. We used the sLORETA-built-in voxel-wise randomization tests with 2000 permutations, based on statistical nonparametric mapping (SnPM). Voxels with significant differences (p < 0.01, corrected for multiple comparisons) between contrasted conditions were located in the MNI-brain www.unizh.ch/keyinst/NewLORETA/sLORETA/sLORETA.htm.

### Statistics

Behavioural data was analyzed using repeated measure and univariate ANOVAs as well as two-tailed t-tests. Neurophysiological data was analyzed by means of mixed effects ANOVAs using the within-subject factors *Go/Nogo* (Go vs. Nogo) and *Time Point* (pre vs. post) and the between-subjects factor *Group* (Neurofeedback vs. waiting list controls). When necessary, the factor *Electrode* was used as an additional within-subject factor. Greenhouse-Geisser correction was applied and post-hoc tests were bonferroni-corrected when necessary. All variables were normally distributed as indicated by Kolmogorov-Smirnov tests (all z < 1.05; p > 0.2).

### Ethical standards

The authors assert that all procedures contributing to this work comply with the ethical standards of the relevant national and institutional committees on human experimentation and with the Helsinki Declaration of 1975, as revised in 2008.

## Additional Information

**How to cite this article**: Bluschke, A. *et al*. The neuronal mechanisms underlying improvement of impulsivity in ADHD by theta/beta neurofeedback. *Sci. Rep.* **6**, 31178; doi: 10.1038/srep31178 (2016).
